# Cardioprotective effect of acupuncture for percutaneous coronary intervention-related myocardial injury in patients with coronary artery disease: A protocol for systematic review and meta-analysis

**DOI:** 10.1097/MD.0000000000020135

**Published:** 2020-05-15

**Authors:** Cong Chen, Xue-ying Zhu, Kun Zhou, Dong Li, Qian Lin

**Affiliations:** a Dongzhimen Hospital, Beijing University of Chinese Medicine, Beijing; b Shandong University of Traditional Chinese Medicine, Shandong, Jinan; c Dongfang Hospital, Beijing University of Chinese Medicine, Beijing, China.

**Keywords:** acupuncture, myocardial injury, PCI, protocol, systematic review

## Abstract

**Background:**

Although patients with coronary artery disease (CAD) rely increasingly upon percutaneous coronary intervention (PCI), this therapy causes subsequent the complications of myocardial injury. Acupuncture safely protects the heart from ischemic injury; however, the efficacy of acupuncture for periprocedural myocardial injury after PCI remains unclear.

**Methods:**

Seven databases in English and Chinese including PubMed, Web of Science, Cochrane Library, Embase, Chinese Biomedical Literature Database, Chinese National Knowledge Infrastructure, and Wanfang Database will be searched. Randomized controlled trials (RCTs) that use acupuncture to treat PCI-related myocardial injury in patients with CAD, regardless of blinding. The crossover randomized trials will be included, but only the pre-crossover data will be analyzed to avoid carryover effects. We will exclude non-RCTs, qualitative studies, uncontrolled clinical trials, and laboratory studies. The measurement of concentration of cardiac troponin (T or I) and MB isoenzyme of creatine kinase will be used as primary outcome. Postprocedural cardiac function and the major adverse cardiac/cerebrovascular event rate will be assessed as secondary outcome. Relevant data were collected independently by 2 reviewers and the third reviewer was responsible for resolving discrepancies through discussion. The Review Manager V.5.3.3 s will be used to perform the data synthesis and subgroup analysis.

**Discussion:**

This systematic review and meta-analysis would provide convincing evidence of various types of acupuncture that specifically focuses on cardioprotective effect of acupuncture on PCI-related myocardial injury.

**Registration:**

Open Science Framework (OSF) registries (osf.io/n2e6t) with the registration DOI: 10.17605/OSF.IO/79H2E.

## 1 Introduction

In patients presenting with coronary artery disease (CAD), percutaneous coronary intervention (PCI) reduces mortality when compared with the alternative therapy like fibrinolysis.^[1]^ However, many cardiac complications of PCI, such as acute thrombosis formation, ischemia reperfusion injury, reperfusion arrhythmia, can independently cause subsequent myocardial injury and cardiac insufficiency.^[2]^ Defined evidence demonstrate the correlation of increased cardiac enzyme release after PCI with delayed mortality.^[3]^ Troponin and MB isoenzyme of creatine kinase (CKMB) elevations after PCI are associated with increased 1-year mortality rates.^[4]^ The periprocedural myocardial injury after PCI still remains to be resolved.

Acupuncture is one of the significant therapeutic modalities of Traditional Chinese Medicine (TCM) and has been accepted worldwide as a promising alternative therapeutic approach in treating diseases. In recent years, many clinical trials demonstrate the cardioprotective efficacy of acupuncture in treating heart disease.^[5]^ Previous studies have shown that transcutaneous electrical acupoint stimulation could regulate the function of autonomic nervous system function, enhance the activity of vagus nerve, and protect myocardial tissue.^[6]^ Currently, whether acupuncture attenuates periprocedural myocardial injury after PCI need to be exhaustively investigated. There are increasing randomized controlled trials (RCTs) testing the cardioprotective effect of acupuncture in treating PCI-related myocardial injury. However, the limitations of these RCTs are relatively small patient sample size. Accordingly, this report presents a protocol for a systematic review of the cardioprotective effect of acupuncture in treating PCI-related myocardial injury in patients with CAD.

## 2 Methods

### 2.1 Study registration

This study has been registered in the Open Science Framework (OSF) registries (osf.io/n2e6t) with the registration DOI: 10.17605/OSF.IO/79H2E. This protocol adheres to the Preferred Reporting Items for Systematic Reviews and Meta-Analyses Protocols (PRISMA-P) statement guidelines.^[7]^

### 2.2 Objectives

The objective of this study is to evaluate cardioprotective effect of acupuncture on PCI-related myocardial injury in patients with CAD through updating relative RCTs. We try to explore whether acupuncture can decrease serum cardiac troponin I and T values and concentration CKMB after PCI.

### 2.3 Inclusion criteria for study selection

#### 2.3.1 Types of studies

We will put all RCTs that use acupuncture to treat PCI-related myocardial injury in patients with CAD, regardless of blinding. The crossover randomized trials will be included, but only the pre-crossover data will be analyzed to avoid carryover effects. We will exclude non-RCTs, qualitative studies, uncontrolled clinical trials, and laboratory studies. tTo minimize publication bias, the study will not be restricted by language or date of publication.

#### 2.3.2 Types of participants

Patients at least 18 years’ old with a diagnosis of stable or unstable angina, or silent ischemia, undergoing nonurgent coronary angiography with the intention to undergo elective PCI, and able to give informed consent, were enrolled.

#### 2.3.3 Types of interventions

The interventions of the including trials will be acupuncture, which is a needle stimulation that elicits deqi sensation by penetrating the skin. We will include all the methods of stimulation, such as electroacupuncture, pharmacopuncture, thread-embedding therapy, acupotomy. There is no limitation on the points of stimulation, types of needle, duration of treatment, or number of treatments. The other methods of stimulation and acupoint-related interventions that do not penetrate the skin with needles (such as moxibustion, acupressure, or laser acupuncture) will be excluded.

#### 2.3.4 Types of outcome measures

The primary end points are:

1. cTn(I or T)
2. CKMB

The secondary end points are:

post-procedural cardiac function
the major adverse cardiac/cerebrovascular event rate.

### 2.4 Data source and search strategy

#### 2.4.1 Data source

Two reviewers systematically searched 4 electronic databases PubMed, Web of Science, Cochrane Library, Embase, and 3 Chinese literature databases, which are Chinese Biomedical Literature Database, Chinese National Knowledge Infrastructure, and Wanfang Database. And the language was not limited. Our meta-analysis only selected the most recent publication, when the same study had been published in different journals or years. If different studies were done and published by same researchers, all studies were selected in the meta-analysis.

#### 2.4.2 search strategy

The main keywords are: acupuncture, percutaneous coronary intervention, coronary artery disease, randomized controlled trials. Details of search strategy in PubMed are as follow:

#1 (“acupuncture” [MeSH Terms]) OR (“electro-acupuncture” [Title/Abstract]) OR (“needling” [Title/Abstract]) OR (“pharmacopuncture” [Title/Abstract])OR (“acupotomy” [Title/Abstract])

#2 (“Percutaneous coronary intervention” [MeSH Terms]) OR (“PCI” [Title/Abstract]) OR (“Coronary Intervention, Percutaneous” [Title/Abstract]) OR (“Revascularization, Percutaneous Coronary” [Title/Abstract]) OR (“Coronary Revascularization, Percutaneous” [Title/Abstract])

#3 (“Coronary artery disease” [MeSH Terms]) OR (“Artery Disease, Coronary” [Title/Abstract]) OR (“Disease, Coronary Artery” [Title/Abstract]) OR (“Arterioscleroses, Coronary” [Title/Abstract]) OR (“Coronary Atheroscleroses” [Title/Abstract])

#4 (“Randomized, controlled trial” [MeSH Terms]) OR (“Randomized controlled trial∗” [Title/Abstract]) (“clinical study” [Title/Abstract]) OR (“Clinical Trial” [Title/Abstract]) OR (“Controlled study∗” [Title/Abstract]) OR (“Controlled Trial∗” [Title/Abstract])

#1 AND #2 AND #3 AND #4

### 2.5 Data collection

**Figure 1:**
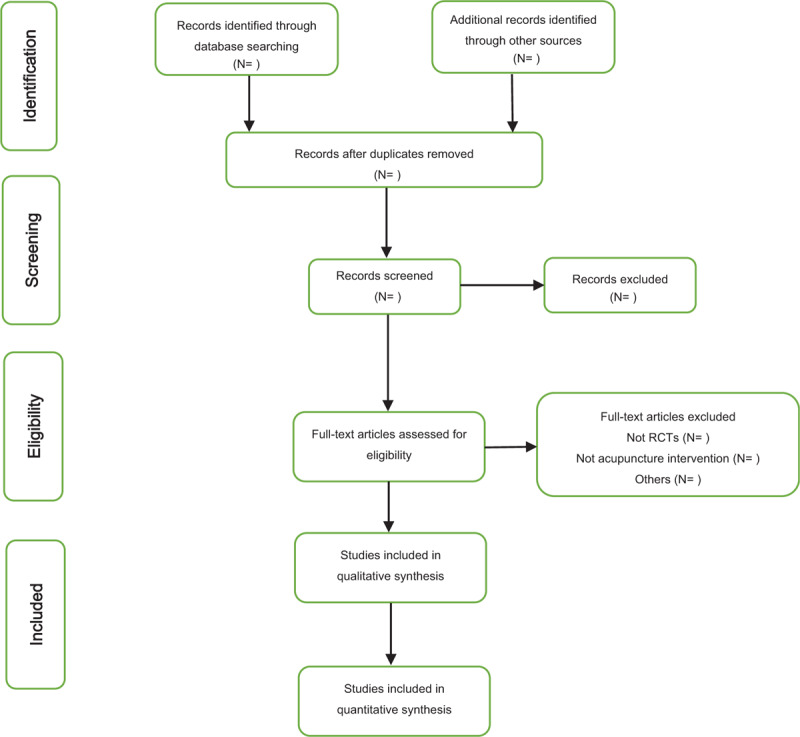
PRISMA flowchart of selection studies.

Two review authors will search potentially relevant studies and duplicate studies will be deleted and remaining eligible studies will be transferred to EndNote X9 software. Through scanning the titles and abstracts of studies, they will further screen the full-text articles to determine which articles may meet the inclusion criteria. If necessary, the third reviewer was responsible for resolving discrepancies through discussion. Details of study selection will be shown as in a PRISMA flow diagram (Fig. 1).

Relevant data were collected independently by 2 reviewers and the third reviewer was responsible for resolving discrepancies through discussion. Standardized predefined form was used including 4 parts: basic information: title, the first author, year of publication, and corresponding address; study characteristics: acupuncture and control intervention, number of sessions, duration, acupuncture points; the characteristics of participants: sample size, gender distribution, mean age and race; results of the study: outcome measures. When there were missing, errors, or ambiguous information in included studies, we will contact original authors of the study. If the author failed to answer the questions, the insufficient information would be neglected or the study would be excluded. Different opinions between reviewers were resolved by communicated with others reviewers.

### 2.6 Data synthesis and statistical analysis

All the statistical analyses were performed using Cochrane Program Review Manager Version 5.3 (Cochrane Collaboration, Oxford, UK). We used *I*^2^ test and *χ*^2^ test to evaluate the heterogeneity of included RCTs, which was to estimate the discrepancy across studies (When *I*^2^ values <50% and *P* > .10, there was not obvious heterogeneity). Random-effect model was utilized when heterogeneity was obvious, otherwise fixed-effect model was used. The risk ratio and the mean difference were used to represent binary variable and continuous variables, respectively, with a 95% confidence interval. Constructing a Funnel graph was used to detect publication bias.

### 2.7 Quality assessment

We assessed the quality of involved RCTs by using the tool of Cochrane Collaboration. Two reviewers who achieved date extraction separately evaluated the risk of bias in involved studies. Scoring dissent was decided by the discussion between authors. Several sources of bias consist of Cochrane Collaboration tool, including Random sequence generation, Allocation concealment, blinding of participants, and personnel blinding of outcome assessment, incomplete outcome data, selective reporting, anything else, ideally prespecified, and others. The reviewers would mark every category according to the available information and there are 3 standards, which are HIGH risk (H), low risk (L), and unclear risk (U). H and L risk of bias can be indicated by judging “yes”’ and “no,” when the characterization was not enough to judge the risk of bias, “unclear” was used. And the study was determined high risk of bias if it received (H) in ≥2 or (U) in ≥3 criteria.

### 2.8 Sensitivity analysis

We will perform sensitivity analyses to assess the robustness and reliability of results. The methods are changing different methods of analysis (random-effects model or fixed-effect model) and eliminating each of the included studies one by one and then combine the effect quantities.

### 2.9 Ethics

Ethical approval is not necessary as all patients provided written consent.

## 3 Discussion

Over the past few decades, the advanced PCI has been proved that it's a better and safer therapeutic procedure than fibrinolysis therapy with minimal procedural complications. But previous investigations have clearly stressed the adverse prognosis of patients undergoing elective PCI, which might therefore reduce the beneficial effects of coronary revascularization itself.^[8,9]^ Myocardial injury is detected when blood levels of sensitive and specific biomarkers such as cTnI or the are increased.^[10]^ The components of the contractile apparatus of myocardial cells are cardiac troponin I and T. They are expressed almost exclusively in the heart. Several studies demonstrated that PCI-related elevations of these biomarkers in the blood reflect injury leading to necrosis of myocardial cells.^[11]^ Previous studies demonstrated the adverse prognosis of patients with CK-MB increases exceeding 5 times (or even 8 times) the upper limit of normal.^[8]^ Stent and atheroablative procedures may confer a higher risk of CK-MB release than PTCA and combined procedures may increase the risk even further.^[12]^ The solid predictor of PCI-related cardiovascular events is cTnI and CK-MB release, which is the reason that was chosen as the primary end point in the present study.

There is evidence that supports the potential myocardial protective effects of acupuncture by inhibiting the elevation of cTnI and alleviating cardiac ischemia-reperfusion injury in adult patients undergoing heart valve replacements.^[5]^ However, some previous published studies always have the several limitations including the number of patients was relatively small and incomplete demographic data. Therefore, it is still uncertain whether acupuncture is cardioprotective effect for PCI-related myocardial injury in patients with CAD, partially due to a lack of high-quality systematic reviews. This systematic review and meta-analysis would provide convincing evidence of various types of acupuncture that specifically focuses on cardioprotective effect of acupuncture on PCI-related myocardial injury.

## Author contributions

**Conceptualization:** Cong Chen, Xue-ying Zhu

**Funding acquisition:** Qian Lin, Dong Li

**Methodology:** Cong Chen, Xue-ying Zhu

**Project administration:** Kun Zhou

**Writing – original draft:** Cong Chen

**Writing – review & editing:** Cong Chen
